# Novel Amoxicillin-Loaded Sericin Biopolymeric Nanoparticles: Synthesis, Optimization, Antibacterial and Wound Healing Activities

**DOI:** 10.3390/ijms231911654

**Published:** 2022-10-01

**Authors:** Shaimaa E. Diab, Nourhan A. Tayea, Bassma H. Elwakil, Abir Abd El Mageid Gad, Doaa A. Ghareeb, Zakia A. Olama

**Affiliations:** 1 Botany and Microbiology Department, Faculty of Science, Alexandria University, Alexandria 21568, Egypt; dalia.diab9@gmail.com (S.E.D.); nourahmed546899@gmail.com (N.A.T.); zakiaolama52@gmail.com (Z.A.O.); 2 Medical Laboratory Technology Department, Faculty of Applied Health Sciences Technology, Pharos University in Alexandria, Alexandria 21500, Egypt; 3 Applied Entomology Department, Faculty of Agriculture, Alexandria University, Alexandria 21545, Egypt; abir_gad@yahoo.com; 4 Biological Screening and Preclinical Trial Lab, Biochemistry Department, Faculty of Science, Alexandria University, Alexandria 21568, Egypt; d.ghareeb@yahoo.com

**Keywords:** sericin/propolis/Amoxicillin nanoparticles, novel antibacterial agent, wound healing

## Abstract

Infected wounds are a major threat among diabetic patients. Technological advancements are currently increasing the number of new adjunctive therapies that may be potent agents for speeding recovery, lowering the amputation rate and limiting infection recurrences. A novel formula with promising antibacterial activity, namely sericin/propolis/Amoxicillin nanoparticles, was assessed as a potent treatment of infected wounds in normal and diabetic rats. Skin wound healing efficiency was assessed through wound healing scorings, bacterial load assessment and histological examinations. It was revealed that upon using sericin/propolis/Amoxicillin nanoparticles, complete wound healing was successfully achieved after 10 and 15 days postinjury for nondiabetic and diabetic rats, respectively. However, the bacterial load in the induced infected wounds was extremely low (0–10 CFU/mL) after 15 days post-treatment. The histological studies revealed that the dermis was more organized with new matrix deposition, and mature collagen fibers were observed among the treated animal groups. The present study is the first preclinical study which reported the importance of silk sericin in the form of nano-sericin/propolis loaded with Amoxicillin as an effective treatment against bacterial wound infections.

## 1. Introduction

Diabetic wounds are considered an major cause of amputations in diabetic patients [1]. The wound healing process is usually complicated, even in nondiabetic patients [2]. The normal wound healing process includes three typical phases, namely inflammation, proliferation and remodeling [3]. The mechanism of poor wound healing in diabetic patients is still under extensive investigations; however, diabetes can involve some deadly complications, e.g., neuropathy, impaired angiogenesis, hypoxia and the stimulation of reactive oxygen species (ROS) [4]. Two key aspects are included in the wound healing process, firstly, to hinder the wound infection, and secondly, to retain a moist environment around the wounded area [2]. Several techniques have been used to combat bacterial infections in diabetic wounds, namely (1) glycemic control, (2) nutritional support, (3) offloading the extremity, (4) treatment of infection, (5) adequate and timely debridements, (6) appropriate topical wound care, etc. Wound infection may lead to improper collagen deposition, fester at the wound site and delaying of the wound healing process [5]. The progressive emergence of multidrug-resistant (MDR) bacteria (caused by the excessive use of antibiotics) is a major obstacle hindering the appropriate wound healing mechanism [1], with *Staphylococcus aureus* (*S. aureus*) and *Pseudomonas aeruginosa* (*P. aeruginosa*) being the major causes of bacterial wound infections [6]. The pharmaceutical industry’s efforts to generate new highly potent antibacterial agents, with novel mechanisms of action to combat MDR bacteria, have weakened dramatically over the last three decades for economic, scientific or strategic reasons [7,8].

Sericin is a natural hydrophilic protein produced by silkworms (*Bombyx mori*) and it ideally constitutes up to 30% of the total silk protein. Sericin has gained researchers’ attention due to its significant physical, chemical and multifunctional biological properties, namely antibacterial, antioxidant and anti-inflammatory effects. It is a biodegradable, biocompatible and nonimmunogenic protein-based biomacromolecule [9] that has attracted many researchers to employ it as a biomaterial for biomedical and pharmaceutical applications, including wound dressings, drug delivery and skin repairing (as a result of its beneficial effect on fibroblasts and keratinocytes) [10]. On the other hand, propolis possesses a good antibacterial ability, rendering it a good candidate for promoting the wound healing process. Propolis is a resinous natural substance composed of bees’ salivary secretions, beeswax and plants exudates [11,12]. Propolis exhibited a wide spectrum of activity (antimicrobial, antiviral and anti-inflammatory) as a result of the presence of flavonoids, steroids, phenolic acids (and their ester derivatives) and amino acids as major compounds in propolis extracts [13]. Moreover, propolis proved a considerable success in enhancing wound dressings’ efficacy in several aspects [14,15].

Biopolymeric nanoparticles have a wide range of applications. Many protein nanoparticles are easy to prepare and can be modified to meet specific requirements such as the vesicle size, shape and improved drug entrapment efficiency. Protein nanoparticles have a wide range of applications due to their known biocompatibility and safety [16].

The aim of the present work was to synthesize a novel biopolymeric nanoformula of sericin and propolis, loaded with a commonly known antibiotic. Statistical optimization was applied to enhance the antibacterial activity and the nanoparticles’ physical characteristics. The optimized nanoformula was applied as a potent treatment against infected diabetic and nondiabetic wounds.

## 2. Results and Discussion

### 2.1. Qualitative, Quantitative and SDS-PAGE Analyses of the Extracted Sericin

Six degumming methods were investigated for the extraction of silk sericin; data revealed that the most effective degumming method used was alkali/high-temperature degumming, with the highest protein content (4.90 g/dL), followed by high-temperature and alkali/high-temperature/high-pressure degumming methods (4.60 and 4.58 g/dL, respectively) (Table 1). The highest A-ratio was noticed with the alkali/high-temperature degumming method (1.77), which was the closest to the ideal value of 1.8 (Table 1).

Similarly, Allardyce et al. [17] reported that the most commonly used and favored method of degumming involves boiling the cocoons in an alkaline solution (alkali/high temperature degumming) because it can extract a high level of sericin content in a short time. Gupta et al. [18] mentioned that the higher the A-ratio, the better the quality of sericin, which was noticed with the standard sericin sample (1.73), followed by sericin extracted from silk fabric, sericin extracted from cocoons and sericin extracted from silk waste (1.35, 1.25 and 1.09, respectively).

**Figure 1:**
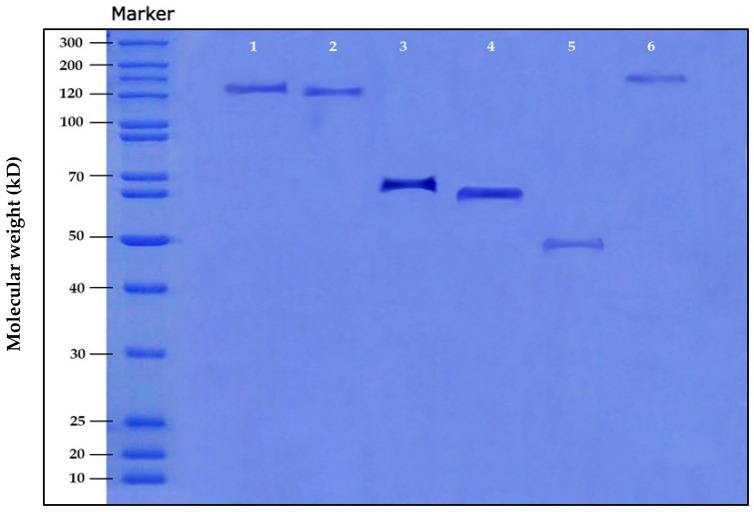
Sodium Dodecyl Sulfate Polyacrylamide gel electrophoresis (SDS-PAGE) chromatogram of the different protein extraction methods (Lane 1: High temperature/high pressure (HTHP), Lane 2: High temperature, Lane 3: Alkali/high-temperature degumming, Lane 4: Alkali/high-temperature/high-pressure degumming, Lane 5: Acid-degumming and Lane 6: Urea buffer).

The SDS-PAGE technique was used to assess the molar mass distribution differences among the various extraction methods under test. Figure 1 shows the sericin molar mass variations through the different extraction methods. Alkali/high-temperature-extracted sericin has a molecular weight of ≈ 70 KDa. Jo et al. [19] reported that the protein content and the molecular weight of silk sericin vary depending on the degumming conditions. According to the previous results, the alkali/high-temperature degumming method was chosen for further analyses.

### 2.2. Amino Acid, X-ray Diffraction (XRD) and Fourier Transform Infrared (FTIR) Analyses

In the present work, the amino acid composition indicated a high degree of sericin purity, with little or no contaminants. Sericin consisted mainly of polar amino acids (44.05%), while nonpolar amino acids were present by only 20.05%, which may differ according to the cocoon type [20]. The polar amino acids in the extracted sericin were serine, threonine, proline, isoleucine and cysteine. On the other hand, the positively charged amino acids were lysine, arginine and histadine, while the negatively charged amino acids were aspartic acid and glutamic acid (Table 2). Data revealed that the most prominent amino acid was serine (30.40%), which was in accordance with Ahsan et al. [21].

### 2.3. Sericin/Propolis Nanoparticles (nSE/P) Preparation and Optimization

In a trial to test the successful sericin/propolis nanoparticles’ formation, different sericin concentrations and stirring times of the reaction mixture were investigated. The data in Table 3 revealed that trial 3 reported the most potent activity (a sericin percentage of 30% and a 45 min stirring time) and lower or higher ratios showed inferior results (Table 4 and Table 5). The physical characteristics of the formulated nano-sericin/propolis proved its stability and high homogeneity, with a +39.3 mv zeta potential, 116.0 nm zeta size and a 0.24 PDI. The antibacterial activity was in accordance with the observed physical characteristics (the highest positively charged zeta potential and the smallest nanoparticle size showed the highest antibacterial activity against all the tested pathogens). The most resistant bacterial strains (*S. aureus* 2, *K. pneumoniae* 2, *E. coli* 1, *A. baumannii* 2 and *P. aeruginosa* 1) were selected for further analyses.

Kanoujia et al. [22] reported that nanoparticles’ size increases with the increase in the sericin concentration. Nanometric size provides numerous advantages such as better tissue penetration and significantly high cellular uptake [22]. Radu et al. [23] mentioned that sericin polymeric nanoparticles showed potent properties, e.g., low toxicity due to their biodegradable nature. The present study was the first to report the synergistic activity and nanoformulation between sericin and Egyptian propolis extract.

**Figure 2:**
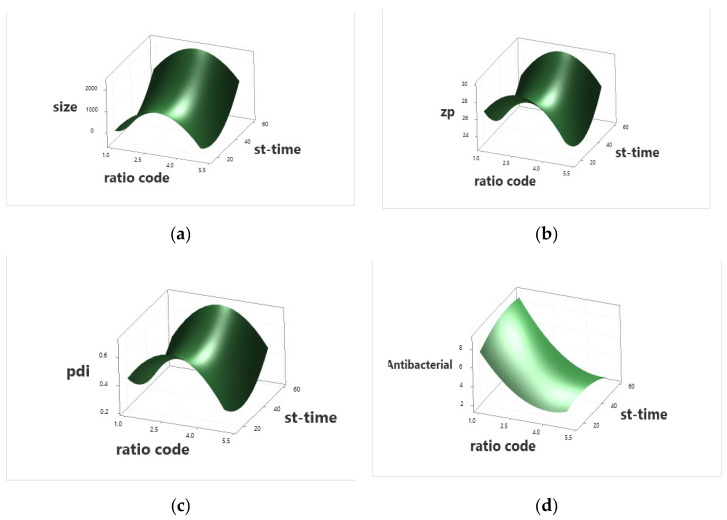
Surface plot of the nanoparticles’ different levels of stirring time and P/SE ratio in response to size (**a**), zeta potential (**b**), PDI (**c**) and antibacterial activity (**d**).

Statistical analysis of the independent variables proved the significance of sericin concentrations and the stirring time of the reaction mixture on the studied responses (Table S1, Equations (1)–(4) and Figure 2). However, the data proved that the interaction between the independent variables was insignificant for the zeta potential (Table S1). The regression model and equations are represented in Table S2. The dependent variables’ equations are mentioned (Equations (1)–(4)).

(1) Zeta size (nm)=−137.929−1.30×A+25.863×B

(2) Zeta potential (mV)=27.10−0.021×A+0.016×B

(3) PDI=0.48−0.0009×A+0.0012×B

(4) Antibacterial activity (mm)=11.852−0.147×A−0.0001×B

where A is the sericin percentage and B is the stirring time.

### 2.4. The Combined Action of the Tested Antibiotics and the Optimized Nanoformula

**Figure 3:**
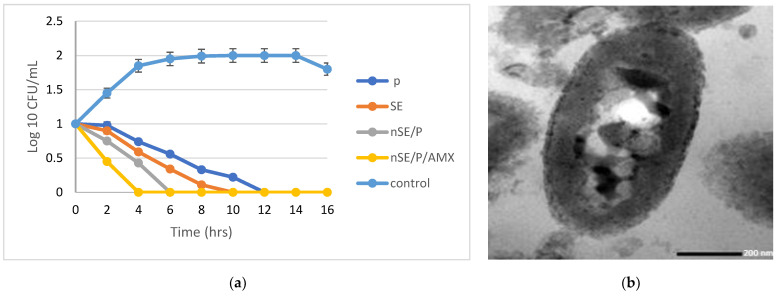
Time kill curve of the bacterial cells treated with SE, P, nSE/P and nSE/P/AMX, one at a time (**a**). Transmission electron micrograph of nSE/P/AMX-treated cells (**b**).

Different antibiotics were combined one at a time with the newly prepared sericin/propolis nanoparticles (Table 5). The highest synergistic activity was observed with Amoxicillin. Different drug concentrations were investigated. The optimum drug concentration that led to maximum entrapment efficiency was 10 mg/mL, and by increasing the drug concentration, the entrapment efficiency decreased (98.2 and 52.4% while using 20 and 30 mg/mL of Amoxicillin, respectively). A one-way ANOVA study proved that the significance of the statistical design with a *p*-value equaled zero and the regression equaled 99.98 (Table S3 and Figure 3). *Pseudomonas aeruginosa* was the most resistant strain and hence was chosen for further analyses through MIC, MBC, MIC index, time kill curve and transmission electron microscope studies.

Data also revealed that a significant reduction in the bacterial growth was noticed after 2 h of incubation with sericin/propolis/Amoxicillin nanoparticles (nSE/P/AMX), and complete eradication of the bacterial growth was noticed after 4 h of incubation, with significant stability (Figure 3a). Moreover, the MIC value of the combined nanoparticles reached 1 µg/mL with a bactericidal effect (Table 6). Further evaluation was confirmed by the transmission electron microscope study, which showed a major synergistic effect between sericin, propolis and Amoxicillin in the nSE/P/AMX formula that led to an enhanced antibacterial activity. The results presented in Figure 3b indicated the leakage of proteinaceous and other intracellular components after the treatment with the tested formula. The observed result may have resulted from the polycationic action of the sericin nanoparticles and their interaction with the negative charge on the bacterial cell surface. Thus, the cell membrane was disrupted, leading to intracellular compound leakage and cell death [24]. Furthermore, Deryabin et al. [25] reported that the polyphenols of propolis interact with many microbial proteins, through the formation of hydrogen and ionic bonds, and thus alter the three-dimensional structure of these proteins and their functionality.

### 2.5. Characterization of the Potent Sericin/Propolis/Amoxicillin Polymeric Nanoparticles

**Figure 4:**
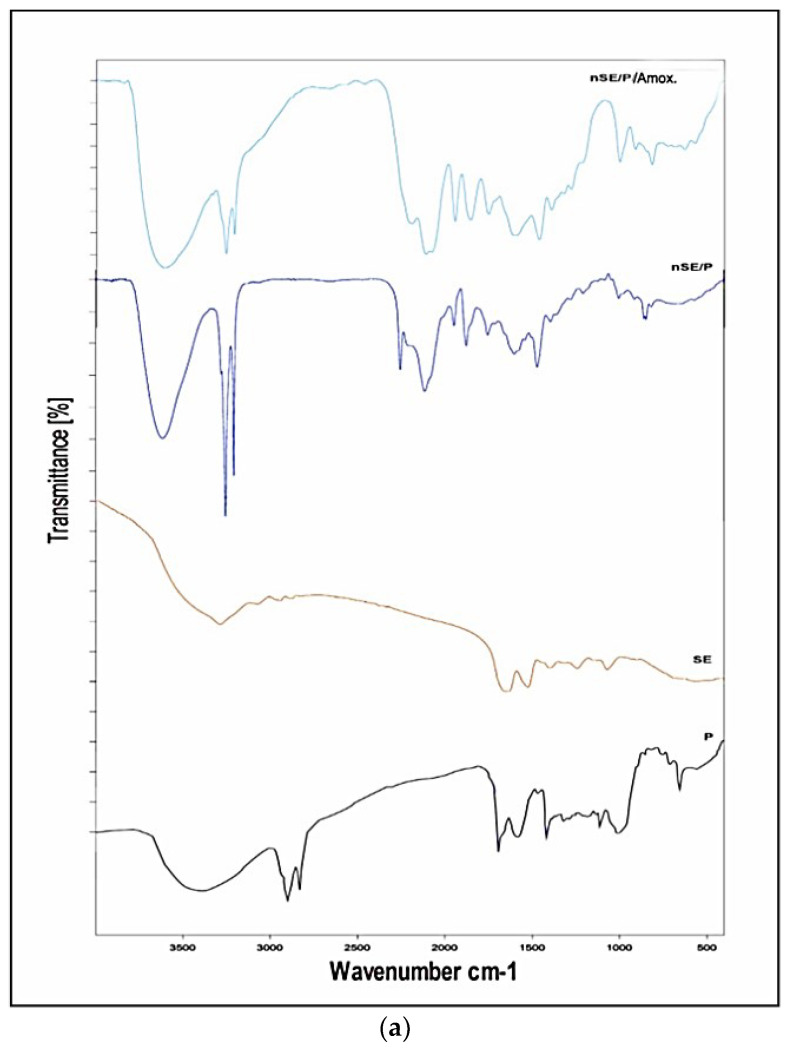

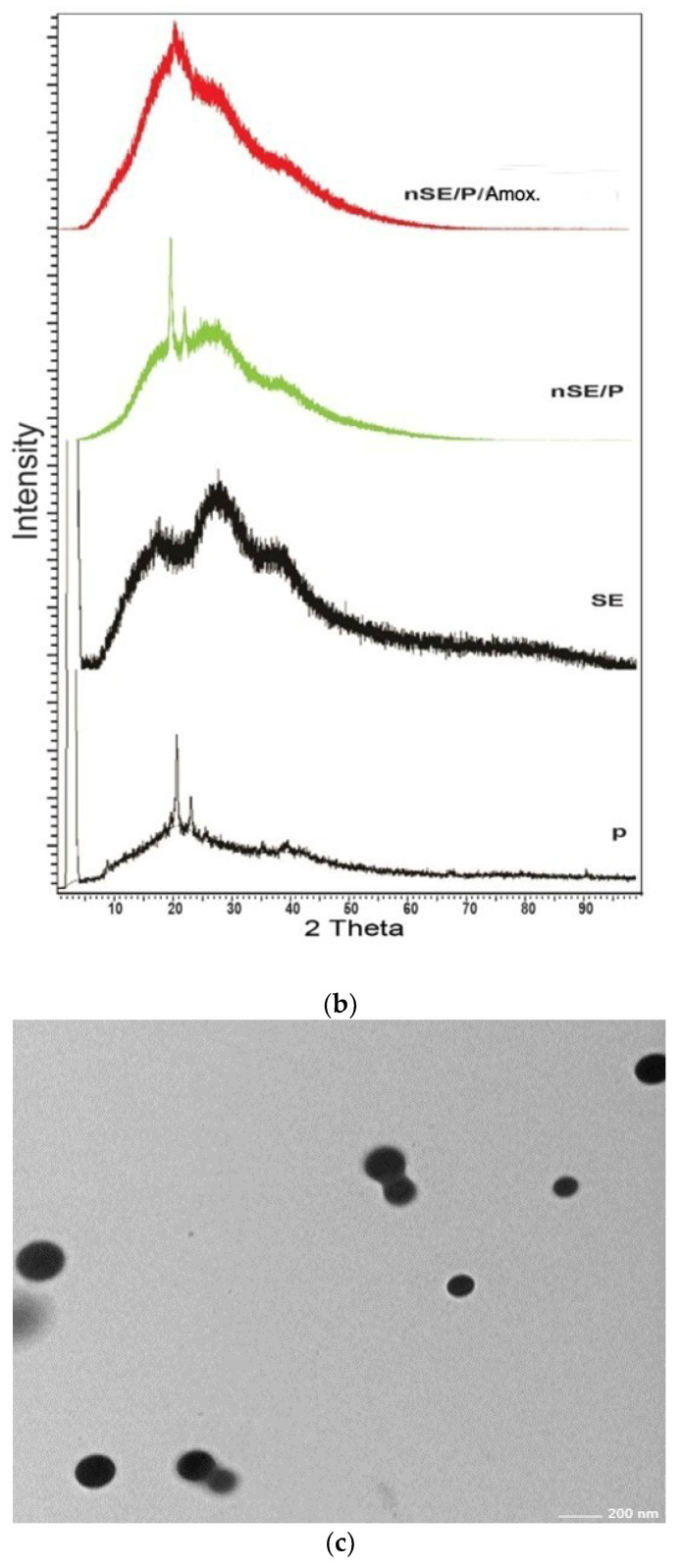
Fourier transform infrared spectra (**a**), XRD of pure sericin, propolis, nSE/P and nSE/P/AMX (**b**) and transmission electron micrograph of sericin/propolis/AMX polymeric nanoparticles (**c**).

The structure and functional groups of sericin, propolis and the promising nanoformula, nSE/P, were characterized using FTIR (Fourier transform infrared) spectroscopy. Figure 4a represents the FTIR spectral details of sericin, propolis, sericin/propolis nanoparticles and nSE/P/AMX. Notably, it has been observed that the IR spectra of sericin showed peaks in the regions of 3000–3500 cm^−^^1^, which were associated with N–H stretching vibrations of amide bonds; Amide I, II and III were detected subsequently at 1651, 1522 and 1241 cm^−^^1^ due to the stretching vibration of the C=O, which was significant for determining the protein structure [26]. The absorption band obtained at 1265 cm^−^^1^ for nSE/P was attributed to P=O and P–C stretching. Furthermore, other bands appeared at 767 cm^−1^ and 1165 cm^−1^, which indicated the overlapping peak of P–O–C and C–O–C, hence, confirming the formation of sericin/propolis nanoparticles from sericin. A new characteristic band appeared at 3420 cm^−1^ due to the amide N–H stretching vibrations that signified the successive incorporation of Amoxicillin and the formation of nSE/P/AMX.

In order to determine the crystalline structure of the prepared nanoformula, nSE/P/AMX, X-ray diffraction analysis was carried out. The samples were thoroughly bombarded with X-rays and the diffraction patterns generated were recorded. Figure 4b shows the X-ray diffraction patterns of sericin/propolis polymeric nanoparticles. The pure sericin gave a characteristic peak located at 2θ = 18.9° and 28° [27]. Propolis gave a characteristic peak located at 2θ = 20° and 22°. XRD of the sericin/propolis nanoparticles showed characteristic peaks at 2θ = 22° and 30°, indicating a shift from the pure sericin peaks around 18.9°, and this shift may be explained by sericin nanoparticles’ formation to control its release at particular target sites, as demonstrated by Suktham et al. [28].

The physical characteristics of the promising nanoformula revealed that nSE/P/AMX has a mean average diameter of 51.50 mm, a PDI value of 0.35 and a zeta potential of +28.00 mV. The morphological examination of the synthesized nanoparticles (nSE/P/AMX) was carried out through transmission electron microscopy. The nanoformula had a vesicle size of 51 nm diameter, was mostly spherical in nature, uniform and aggregated (Figure 4c).

#### In Vitro Drug Release

**Figure 5:**
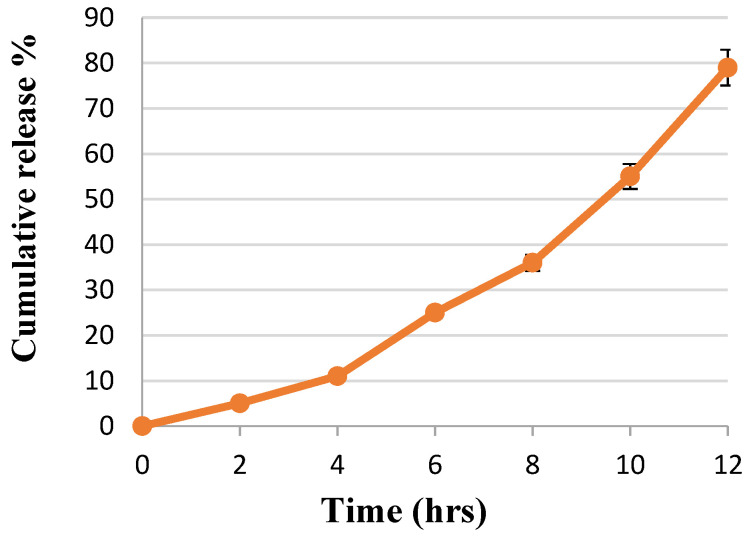
Rate of release of Amoxicillin from the synthesized nSE/P/Amoxicillin.

The main objective of the present study was to test the ability of the novel designed nSE/P/AMX to release the drug load (Amoxicillin) efficiently. The drug release behavior using the dialysis bag method is demonstrated in Figure 5. It was noticed that the prepared nanoformula was able to release the drug load gradually. In the initial release, the prepared nanoformulae showed a significant (*p* < 0.05) retardation release rate. After 2 h, the Amoxicillin cumulative release % reached 5. Then, sustained release occurred due to the diffusion of the drug (Amoxicillin) from the nanoformula. Encapsulation of the drug by sericin/propolis nanoparticles allowed the active prolonged release profile, hence, overcoming the problem of the drug’s short half-life due to the rapid and total accumulative release [29].

### 2.6. In Vivo Studies Using Nondiabetic and Diabetic Rats

#### 2.6.1. Histological Observation of the Negative Control (Wounded Noninfected Nontreated) Rats

**Figure 6:**
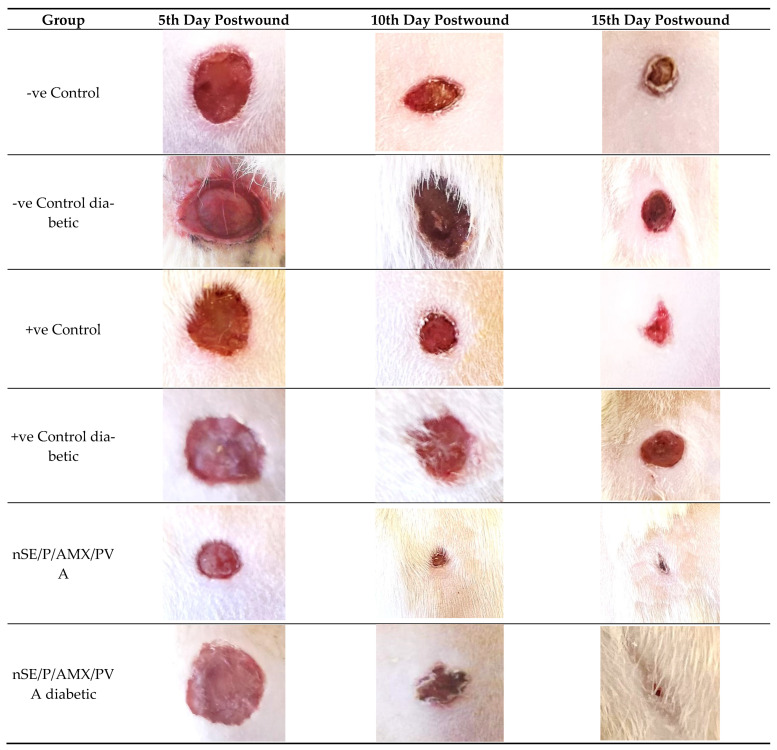
Morphological evaluation of nSE/P/AMX/PVA-treated bacterial wound skin infection in normal rats, showing rapid gradual wound healing at different time intervals, referring to the controls.

**Figure 7:**
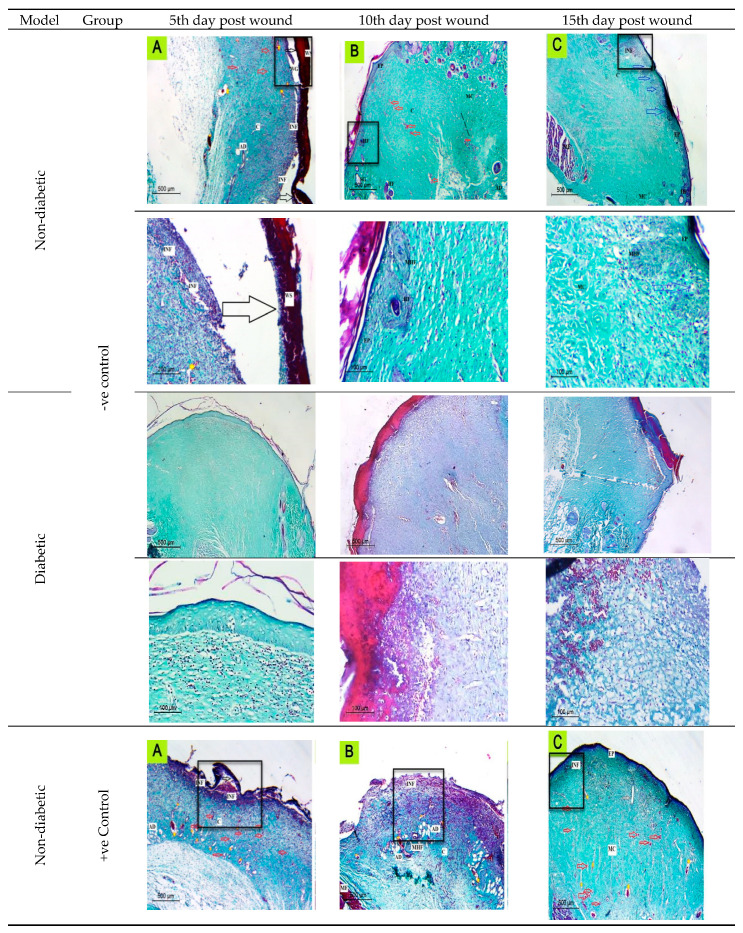

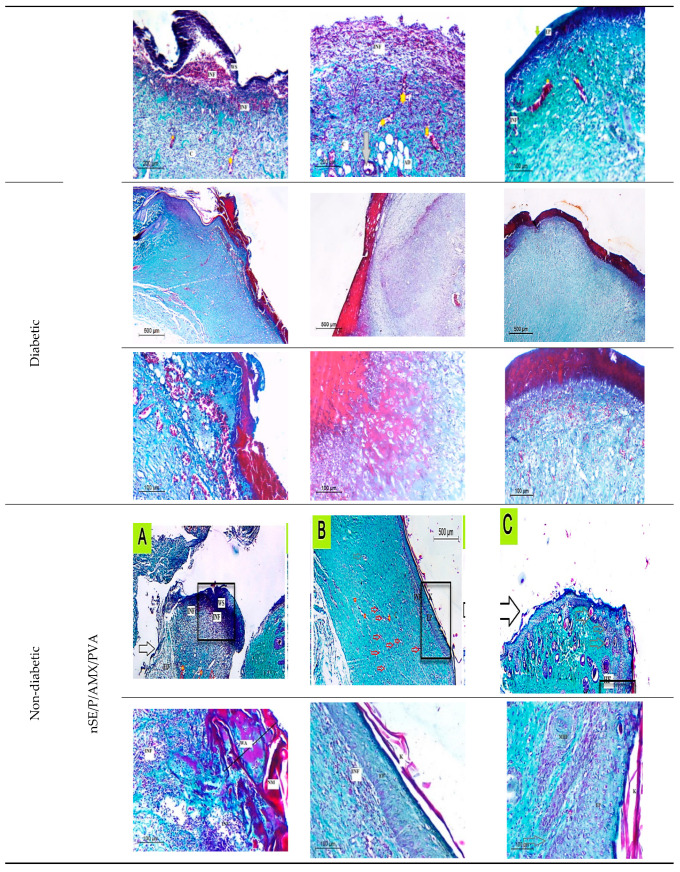

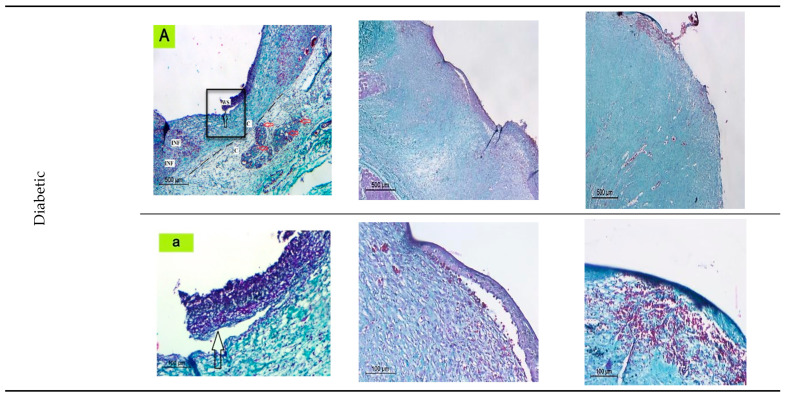
The histological evaluation of skin wounds at different time intervals (5th, 10th, and 15th) indicated regression of the lesions with better epithelialization (blue arrows) and more effective re-organization of the dermis by collagen fiber maturation. Inflammatory cells (INF); Adipose connective tissue; blood vessels (red arrows); Immature collagen (IMC); mature collagen (MC); epidermis (EP); maturating hair follicle (MHF); Wound Gap (WG); wound area (WA); wound scab (WS) dilated blood vessels (Yellow Strikes); Muscle Fibers (MF); detached scabs (black arrows).

**Figure 8:**
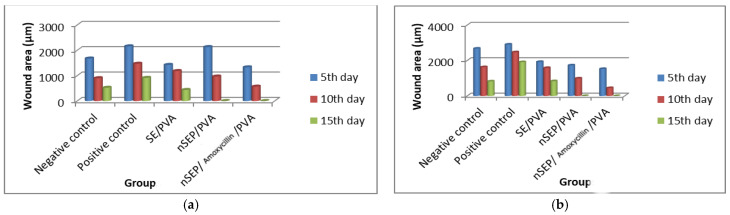
Wound contraction area (**a**): nondiabetic and (**b**): diabetic.

**Figure 9:**
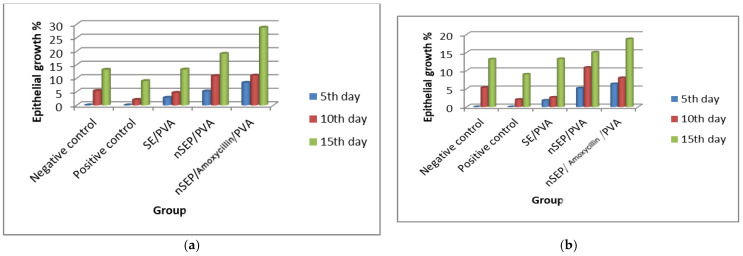
Percentage of epithelial growth using the potent sericin polymeric nanoparticles (**a**): nondiabetic and (**b**): diabetic.

Histological examination of the wounded normal and diabetic rats’ skin at different time intervals revealed that on the fifth day after the rats’ wound induction, skin histological sections showed significant damage in the epidermis and dermis tissue. Inflammatory cell infiltration was also observed in the wound bed, which was dispersed over the dermal middle layer tissue. Furthermore, a large scab was detached from the wound area. Incomplete epithelialization was observed at the 10th day after the rats’ wound induction, and the epidermis of the normal skin surrounding the wound area appeared to be thicker than that of the wound area, as a significant indicator of the healing process. On the 15th day after the rats’ wound induction, a totally healed epithelium was noticed with columnar cells, the dermis layer showed few collagen depositions and the stratum corneum was clearly observed at the most upper layer over the epidermis (Figure 6, Figure 7, Figure 8 and Figure 9).

On the other hand, in diabetic rats at the 10th day after injury, a scab was denoted, there was no epithelium, except on the periphery wound area, and the dermis was full of blood vessels. The epidermis was thrown into the dermis in the form of epidermal tongues in some peripheral areas. On the last interval (15th day after injury), epithelization occurred only at the periphery and was not found in the wound center, which was not covered by epidermis; dermis with collagen fibers at the periphery with extravasated cells were also not found in the wound center (Figure 6, Figure 7, Figure 8 and Figure 9).

#### 2.6.2. Histological Observation of the Bacterial Infected (Nontreated) Wounded Rat Skin

On the 5th day after the infected wound induction (nontreated), sections revealed significant damage in the epidermis and dermis tissue. Furthermore, necrotic material was also observed in the wound gap fill. Inflammatory cell infiltration was also noticed in the wound bed, which was dispersed over the dermal middle layer tissue and was also noticeable under the rebuilt epidermis at the end of the experiment (15th day after the infected wound induction). A big scab encompassed the wounded spot, which disappeared totally after a few days (15th day after the infected wound induction). On the 10th day after the wound induction, incomplete disorganized epithelium with no collagen fibers was noticed, and macrophages and extravasated cells appeared clearly at this stage. After the 15th day after the infected wound induction, a regrowth of the epidermal layer was accomplished to a significant extent. There were numerous dilated blood vessels observed throughout the experiment stages until the last interval, indicating the delayed wound healing stage. The dermis showed few collagen depositions when stained with Masson’s trichrome on the 15th day after the infected wound induction, with more maturation of the epithelium; the basal layer of the epithelium was not arranged normally and a very thick stratum corneum appeared (Figure 6, Figure 7, Figure 8 and Figure 9).

On the other hand, in diabetic rats, the number of blood vessels was more than that of negative control, and no epithelization was noticed on the fifth day after injury. On the 10th day, an epidermal scab was noticed, epidermis was noticed at the junction between the normal and the wounded area, the dermis was disorganized and full of collagen fibers with extravasated cells and blood capillaries, and some dermis areas appeared vacuolated. On the day 15th after injury, a scab was found in complete epithelization, the dermis was disorganized, the outer layers contained fibers, and there were extravasated cells at the periphery under the scab (Figure 6, Figure 7, Figure 8 and Figure 9).

#### 2.6.3. Histopathological Evaluation of SE/PVA Gel Treated Group

On the fifth day after wound induction, no epithelium was denoted, less extravasated cells were present than in the infected nontreated rats; however, the histological picture was more organized than that of the positive control group (the infected nontreated rats’ model) at this stage. On the tenth day, there were traces of wound scab separation with underlying growing epidermis, which covered all the wound area completely with a double to triple thickness of the surrounding normal skin. At the last interval, the epidermal layer was substantially divided into discrete layers. Inflammatory cells were progressively detected and diffused all over the wound area, which disappeared in the last interval, suggesting the start of the next phase. A large number of blood vessels was observed in the dermal layer, some of which were dilated and aligned with disordered immature collagen strands, in order to facilitate the healing process. The blood vessels’ percentage reduced as time passed, demonstrating the usual healing tendency. The commencement of adipose tissue regeneration was visible, but it was still dispersed at the 15th day after the wound induction (Figure 6, Figure 7, Figure 8 and Figure 9).

On the other hand, in diabetic rats at the fifth day after the wound induction, no epithelium was denoted. At the tenth day, epithelial cells were clearly noticed at the wound periphery and were engorged with blood vessels and extravasated cells. At the last interval, complete epithelization and epidermis covering most of the wounded area were noticed. A scab was found, and the dermis extracellular matrix secretions appeared more organized (Figure 6, Figure 7, Figure 8 and Figure 9).

#### 2.6.4. Histopathological Evaluation of PRO/PVA Gel Treated Group

On the fifth day after wound induction, no epithelium was denoted. On the tenth day, epithelial cells were clearly noticed with extravasated cells. At the last interval, the outermost layers were cornified, epithelization was complete, and the epidermis was organized with extracellular matrix and collagen fiber depositions (Figure 6, Figure 7, Figure 8 and Figure 9).

On the other hand, in diabetic rats on the fifth day after wound induction, no epithelium was denoted. On the tenth day, no epithelial cells were clearly noticed with extravasated cells and blood vessels. At the last interval, a scab was present on the center of the wound, epithelization was complete, and the epidermis was organized with extracellular matrix and fiber deposition. The dermal area contained enormous blood vessels (Figure 6, Figure 7, Figure 8 and Figure 9).

#### 2.6.5. Histopathological Evaluation of nSE/P/PVA Gel Treated Group

The first interval findings indicated a huge scab region that had nearly or completely separated from the disordered dermal tissue. The epidermis had been completely obliterated and disturbed. On the 10th day after wound induction, the wound center was covered by a scab, entrapping cellular debris and phagocytes, a thick wound epithelium was noticed at the scab periphery, numerous blood capillaries were noticed under the regenerated epithelium, and the dermis fibers appeared to be perpendicular to the wound epithelium. At the last interval, the epithelium growth continued under the scab with the accumulation of extravasated cells, and the dermis was more organized with new matrix deposition, and mature collagen fibers were denoted by this time interval (Figure 6, Figure 7, Figure 8 and Figure 9).

On the other hand, in diabetic rats at the first interval, many dilated blood vessels were noticed in the epidermis, and the dermis fibers were parallel to the surface with less collagen secretions. At the second interval (10th day after injury), a scab was found, and the dermis was more organized with more collagen secretions. At the last interval, the epithelium was organized with extravasated cells, the dermis was more organized with new matrix deposition, and mature collagen fibers were denoted by this time interval (Figure 6, Figure 7, Figure 8 and Figure 9).

#### 2.6.6. Histopathological Evaluation of nSE/P/Amoxicillin/PVA Gel Treated Group

On the 5th day postwounding, the healing pattern showed the appearance of a large scab attached directly to the remnant dermis. Furthermore, inflammatory cells could be observed at the wound area, whereas the appearance of partial separation of the wound area emphasized the rapid healing pattern. It was fascinating to see how the keratinized epidermis at the last interval had become fully grown, indicating the powerful healing effect of the prepared nanoparticles. The lower dermal layer showed small, dilated blood vessels, which disappeared completely on the 15th day postwounding. There was limited evidence for dermal layer healing. In particular, it displayed little collagen pattern of shredded dermal remnants. Sections exhibited an uneven arrangement of collagen with higher density, as well as an intensive proliferation and dense collagen deposits at the final interval (Figure 6, Figure 7, Figure 8 and Figure 9).

**Figure 10:**
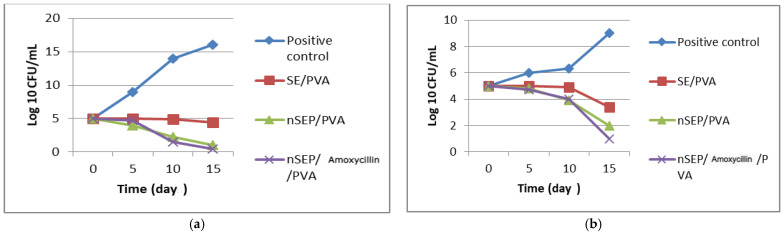
Bacterial load count (**a**): nondiabetic and (**b**): diabetic.

On the fifth day after wound induction in diabetic rats, it was noticed that the wound area was covered with epithelium, with cells not sharply visible. At the second interval, the epithelium at the wound periphery was not noticeable in the center, and the dermis fibers were perpendicular to the surface at the last interval; epithelization was complete on the surface, massive blood vessels were denoted, a scab was present in some areas, there were many collagen depositions on the epidermis and extravasated cells were also noticed under the epithelium (Figure 6, Figure 7, Figure 8 and Figure 9). It is worth noting that nSE/P/Amoxicillin/PVA gel eradicated the bacterial growth (Figure 10).

Topical antimicrobial therapy emerges as an attractive route for the treatment of infectious diseases. A large number of bioactive compounds have been successfully administered via cutaneous administration because of advances in the design of topical and transdermal formulations. The topical antimicrobial therapy is based on the absorption of high drug doses in a readily accessible skin surface, resulting in a reduction in microbial proliferation at infected skin sites. Topical antimicrobials have the following advantages over traditional approaches: a. They are able to escape the enzymatic degradation and rapid clearance in the gastrointestinal tract that occurs during oral administration. b. They alleviate the physical discomfort related to intravenous injection. c. They reduce the possible adverse effects and drug interactions of systemic administrations. d. They increase patient compliance and convenience. e. They reduce treatment costs [30]. In addition, sericin can promote skin keratinocytes’ and fibroblasts’ adhesion and proliferation [31], which makes it favorable for wound dressing and tissue engineering applications [32]. Sericin can efficiently promote wound healing by accelerating collagen deposition and the re-epithelialization of skin tissue [33]. However, sericin has a large amount of disordered structures, resulting in its poor mechanical performance [34]. Thus, crosslinking, blending, or copolymerizing with other substances are often applied to overcome the brittleness of sericin [35]. Hence, the formed nanoformula of sericin/propolis loaded with amoxicillin offers to be a newly potent candidate in combating the drug-resistant wound infections in diabetic and nondiabetic patients.

## 3. Materials and Methods

### 3.1. Bombyx mori Cocoons

The silkworm breed (*Bombyx mori*) selected for the present work was a Bulgarian hybrid (C2 X V2 X kk X H2), disease free, obtained from the Scientific Center on Sericulture, Vratsa, Bulgaria. After hatching, larvae were isolated from the stock culture and were fed with fresh mulberry (*Morus alba* L.) leaves five times per day. The cocoons were dried after 3 days of spinning for further investigations.

### 3.2. Propolis Collection and Extraction

Propolis samples were collected during summer 2020 in Alexandria. The collected samples were kept in sterile dark glass containers until further use. Propolis samples were macerated (30% *w/v*) by ethanol 99% for 48 h at room temperature, with continuous stirring, and then the samples were homogenized for 1 h at 72 °C. Propolis extract was filtered, dried using a rotary evaporator and then the extract was analyzed using GC–MS analysis (reported in our previous publication [36]).

### 3.3. Microorganisms

The bacterial isolates used throughout the present work, namely *Staphylococcus aureus* 1 and 2, *Klebsiella pneumonia* 1 and 2, *Escherichia coli* 1 and 2, *Pseudomonas aeruginosa* 1 and 2, and *Acinetobacter baumannii* 1 and 2, were kindly provided and phenotypically identified by The Microbiology Department’s strain bank of the main University Hospital, Alexandria, Egypt.

### 3.4. Sericin Extraction

Different degumming methods were investigated for silk sericin extraction, namely high temperature and high pressure (HTHP), high temperature, acid-degumming, alkali and high temperature, alkali and high temperature/high pressure, and urea buffer degumming methods, according to Liu et al. [37]. Briefly, in each degumming method, 10 g cocoons were cut into pieces and washed with deionized water. The cocoon pieces were then immersed in the extraction solution. In the alkali and high temperature method, the cut pieces of the cocoons were immersed in 0.02 M Na_2_CO_3_ solution (200 mL) and boiled for 30 min. The silk fibroin was removed by filtration, and the supernatant was dialyzed against deionized water for Na_2_CO_3_ removal for 4 days at room temperature. The filtrate was then freeze-dried for a sericin powder product using a Heto LL-3000 lyophilizer (HetoHolten A/S, Allerod, Denmark).

#### 3.4.1. Sericin Characterization

##### Qualitative and Quantitative Analysis of the Extracted Sericin

The extracted sericin quantity was evaluated through protein content determination by Lowry’s method using bovine serum albumin (BSA) as a standard [18]. Moreover, a qualitative estimation of sericin purity was performed by ultraviolet spectroscopy (D-2750 UV-Vis spectrophotometer, Shimadzu, Singapore), according to the following equation (Equation (5)). When the A-ratio of the extracted sericin sample was above 1.8, then the sample was considered pure [18].

(5) $$A-\mathrm{ratio}=\frac{\mathrm{Sample}\;\mathrm{absorbance}\;\mathrm{at}\;280\;\mathrm{nm}}{\mathrm{Sample}\;\mathrm{absorbance}\;\mathrm{at}\;260\;\mathrm{nm}}$$

#### 3.4.2. Sodium Dodecyl Sulfate-Polyacrylamide Gel Electrophoresis (SDS-PAGE)

The SDS-PAGE analyses of the extracted sericin samples were performed according to Laemmli [37]. Sericin samples (100 µL) were mixed with 100 µL SDS-PAGE sample buffer (2-mercaptoethanol 5%, SDS 2.5%, 0.5 M Tris-HCl of pH 6.8, Bromophenol Blue 0.025% and glycerol 10%) and then boiled for 5 min. The electrode buffer consisted of 125 mM Tris base with 0.96 M glycine and 0.5% SDS. The stacking gel was 5%, while the resolving gel was 12%. The gel was run at 90 V for 4 h. The gel was stained with the stain solution (0.1 g Coomassie blue R-250 dissolved in methanol: acetic acid: water (50:10:40)) for two hours. The stained gel was then destained using the destain solution (methanol, 10% and acetic acid, 7%), three times successively. The molecular weight of each sericin sample was estimated using a molecular weight marker (MWM).

#### 3.4.3. Amino Acid, X-ray Diffraction (XRD) and Fourier Transform Infrared (FTIR) Analyses

The most potent extracted sericin sample was subjected to amino acid, XRD and IR analyses. An ultra-high-performance (UHPLC) hydrophilic liquid chromatography (HILIC) (Eksigent ekspertTM ultraLC 100 system, Dublin, CA, USA)–tandem mass spectrometry (MS) (AB SCIEX instruments, Foster City, CA, USA) method was applied to quantify the amino acids presented in the most potent sericin sample [38]. XRD analysis was used to record the crystallinity index of the extracted sericin, where the sample was mounted on the horizontal axis and the diffracted beam optics were mounted on the 2θ axis [18]. FTIR analysis was applied to the potent sericin sample in order to determine the functional groups using a BRUKER FTIR instrument (the wave number ranged from 4000 cm^−1^ to 4500 cm^−1^) [18].

### 3.5. Sericin/Propolis Nanoparticles’ (nSE/P) Preparation and Optimization

Sericin/propolis nanoparticles’ (nSE/P) novel preparation was performed by varying the sericin concentration and the stirring time of the reaction mixture. The suspensions obtained were subjected to primitive characterization using a Zetasizer (Malvern Zetasizer Nano ZS, Cambridge, UK) to confirm the nanoparticle synthesis.

#### Experimental Design

A two-factor, five-level (2^5^) factorial design was designed to optimize the formulated nSE/P using response surface methodology (RSM), according to the central composite design (CCD). Two factors representing the independent variables were selected, namely the sericin percentage (A) and the stirring time of the reaction mixture (B) (Table 7). The influence of these factors on the response of the formulated nSE/P was investigated, namely the particle size (Y_1_), the zeta potential (Y_2_), the polydispersity index (PDI) (Y_3_) and the antibacterial activity (Y_4_). The Design-Expert 12.0^®^ software package from Stat-Ease was used to implement the experimental designs, optimize and screen most of the parameters that could interfere with the formulated nSE/P using multifactorial CCD. Fisher’s Least Significant Difference (LSD) post hoc test was applied after running the Analysis of Variance (ANOVA), to analyse and identify the pairs of means that were statistically different according to t-values. The mathematical equation that provides an illustration for the response is as follows (Equation (6)):

Y = b_0_ + b_1_A + b_2_B + b_12_AB + b_11_A^2^ + b_22_B^2^ (6)

where Y represents the selected response, b_0_ represents the intercept, and b_1_, b_2_, b_12_, b_11_ and b_22_ were the regression coefficients. A and B were the studied independent factors; AB symbolize the interactions between the main factors, while A2 and B2 indicate the polynomial terms [39].

### 3.6. The Combined Effect of the Tested Antibiotics and the Optimized Nanoformula

Sericin/propolis nanoparticles (nSE/P) encapsulated with different antibiotics (Cephalexin, Colistin, Amoxicillin, Tetracycline, Tegacycline, Chloramphenicol, Ampicillin-sulbactam, Cefotaxime, Amikacin, Gentamicin, Cefoperazone, Azithromycin, Ceftazidim and Cefuroxime, one at a time) were prepared by mixing the optimum nSE/P preparation trial and 10 mg of each tested antibiotic, and then the antibacterial activity of each nanoformula was tested using the disc diffusion method.

The most effective nanoformula was further investigated by using different antibiotic concentrations (10, 20 and 30 mg), one at a time. The nanoformulae suspensions were ultracentrifuged at 25,000× *g* rpm for 20 min to precipitate the formed nanoparticles and then stored at 4 °C in sterile Falcon tubes until further investigations.

The drug entrapment efficiency was calculated for each prepared nanoformula using the following equation (Equation (7)):

(7) $$\mathrm{Entrapment}\;\mathrm{efficiency}=\frac{\mathrm{Amount}\;\mathrm{of}\;\mathrm{drug}\;\mathrm{entrapped}\;\mathrm{within}\;\mathrm{nanoparticles}}{\mathrm{Total}\;\mathrm{amount}\;\mathrm{of}\;\mathrm{initial}\;\mathrm{drug}\;\mathrm{used}}\times 100$$

Moreover, the antibacterial activity of the optimized nSE/P loaded with the most potent antibiotic was investigated by determining the minimum inhibitory concentration (MIC) values and the bacterial lethality curve [13].

### 3.7. Characterization of the Most Promising Nanoformula

FTIR, XRD and transmission electron microscopic (TEM) examinations of the most promising nanoformula were performed according to Elnaggar et al. [13] in order to assess the functional groups, the nanoparticles’ crystallinity and ultrastructure, and the size and shape of the most promising nanoformula, respectively. The experiments were carried out at the Central Lab, Faculty of Science, Alexandria University, Egypt.

### 3.8. In Vivo Study on the Wound Healing Efficiency of the Synthesized Nanoparticles in Normal and Diabetic Rats

#### 3.8.1. Fabrication of PVA Gel Combined with the Optimized Nanoformula

Sericin/poly vinyl alcohol (PVA) gel was prepared, according to Siritienthong et al. [40], with some modifications. Briefly, PVA (6% wt) was dissolved at a constant concentration of under constant stirring at 80 °C for 4 h. Sericin solution (3% wt), 2% PVA solution and glycerin solution (1% wt) were mixed for 1 h at room temperature to prepare the sericin/PVA treatment gel. The mixture was then poured into a sterile Petri dish and frozen at −20 °C. Propolis/PVA (PRO/PVA), nSE/P/PVA and nSE/P/Amoxicillin/PVA gels were prepared in accordance with the sericin/PVA preparation method, using the same conditions [40].

#### 3.8.2. Animal Modeling

The animals were maintained for 2 weeks under animal ethical guidelines before the grouping. One hundred and twenty (120) male albino rats (*Rattus norvegicus albinus*), 4 months old and with an average body weight of 180 ± 30 g, were assigned to six groups. Each group (20 rats) was divided into two subgroups (10 rats/each), one for the diabetic and the other for the nondiabetic models, and kept in separate cages under average conditions of temperature (25 ± 2 °C) and humidity for 30 days, inside an adequately ventilated room. For the diabetic induction, each rat was anesthetized with 10% chloral hydrate through intraperitoneal injection (0.03 mL/g weight).

Diabetes mellitus (type 1) induction was performed according to Furman [41]; a single dose of Streptozotocin (65 mg/kg) was injected intraperitoneally, then after 7 days, the blood glucose level was tested and when the blood glucose levels were >150 mg/dL, the rats were considered diabetic.

Then, an artificial wound (1 cm diameter) was created on the back of each rat after removing the dorsal hair. Then, 50 μL of *P. aeruginosa* (1.0 × 10^7^ CFU/mL) was inoculated to infect the wounds.

The rats were subjected to a regular 12 h light/12 h dark cycle and were allowed free access to food and water ad libitum, and both the diabetic and nondiabetic rats were divided into six groups according to their treatment regimen as follows:

- Group I: Assigned as a negative control, with neither infection nor treatment regimen.
- Group II: Assigned as a positive control, infected with MDR bacteria with no treatment regimen.
- Group III: MDR bacterial infected rats treated with sericin/PVA.
- Group IV: MDR bacterial infected rats treated with propolis/PVA.
- Group V: MDR bacterial wound infected rats treated with a placebo (nSE/P) nanoparticles/PVA.
- Group VI: MDR bacterial wound infected rats treated with the final nanoformula/PVA.

Topical treatment for all rat groups (nondiabetic and diabetic) was initiated by receiving 0.5 g of each treatment once/day for 10 days. They were applied until the complete healing of the wounds in one of the experimental groups was observed. The progressive changes in the wound area were monitored, and the wound area was measured every day. The percentage of wound contraction was calculated by the following formula (Equation (8)):

(8) $$\mathrm{WC}=\frac{S0-S}{S0}\times 100$$

where S0 was the initial wound area and S was the wound area in a specific day [40].

#### 3.8.3. Bacterial Load Assessment

The rats were executed at predetermined intervals (5, 10 and 15 days after wound treatment), and the wound surface was softly cleaned with 70% ethanol and excised. After weighing the tissue, it was homogenized in 1 mL of phosphate buffer (PBS) under aseptic conditions. Before plating onto blood agar, the homogenized tissue was serially diluted with PBS. The plates were incubated for 24 h at 37 °C, and the number of viable cells (CFU/gram tissue) was estimated using Equation (9) at the end of the incubation time [42]:

(9) $$\mathrm{CFU}/\mathrm{gramof}\;\mathrm{tissue}=\mathrm{plate}\;\mathrm{count}\;\left(\frac{1}{\mathrm{dilution}}\right)\times \left(\frac{10}{\mathrm{weight}\;\mathrm{of}\;\mathrm{tissue}}\right)$$

#### 3.8.4. Histological Studies

The excised skin tissue of each group of nondiabetic and diabetic rats was collected and subjected to histological examination. Tissue samples were fixed in 10% formalin to allow good penetration of the fixative for histological examination. The fixed specimens were dehydrated through ascending grades of ethanol. After dehydration, the specimens were embedded in Paraplast. Sections (5 µm thick) were stained with Masson’s trichrome for histological evaluation.

## 4. Conclusions

The results of the present investigation concluded that different sericin extraction methods reported different protein contents; the alkali and high-temperature degumming extraction method showed the highest protein content and purity. The amino acid composition indicated a high degree of sericin purity, with little or no contaminants. The physical characteristics of the formulated nano-sericin/propolis proved its stability and high homogeneity, with a +39.3 mv zeta potential, 116.0 nm zeta size and a 0.24 PDI. Antibacterial activity was in accordance with the observed physical characteristics. A significant reduction in the bacterial growth was noticed after a 2 h incubation with sericin/propolis/Amoxicillin nanoparticles (nSE/P/AMX), and complete eradication of the bacterial growth was noticed after a 4 h incubation, with significant stability. nSE/P/Amoxicillin showed potent wound healing efficiency in normal and diabetic rats, with complete epithelization, massive blood vessels and the presence of collagen depositions on the epidermis; extravasated cells under the epithelium were also noticed. It is worth noting that nSE/P/Amoxicillin/PVA gel eradicated the bacterial growth in the infected wounds. nSE/P/Amoxicillin can be further applied as a novel potent candidate in combating drug-resistant wound infections in diabetic and nondiabetic patients.

## Figures and Tables

**Table 1:** Protein content assessment using different extraction methods.

Extraction Method	Protein Content (g/dL)	A-Ratio
High temperature and high pressure (HTHP)	4.40 ± 0.1	1.34
High temperature	4.60 ± 0.07	1.70
Alkali and high-temperature degumming	4.90 ± 0.09	1.77
Alkali/high-temperature/high-pressure degumming	4.58 ± 0.5	1.65
Acid-degumming	3.70 ± 0.7	1.21
Urea buffer	3.70 ± 0.2	1.20

**Table 2:** Amino acid composition of silk sericin.

Type	Amino Acids	Molar %
Polar	Serine	30.40
	Cysteine	3.05
	Proline	0.80
	Threonine	6.00
	Tyrosine	3.80
Nonpolar	Isoleusine	1.40
	Leucine	0.60
	Proline	0.80
	Glycine	12.20
	Phenylalanine	0.40
	Methionine	0.05
	Alanine	4.60
Basic	Histadine	0.90
	Arginine	2.80
	Lysine	10.20
Acidic	Aspartic acid	19.10
	Glutamic acid	4.10

**Table 3:** Optimization of the physical characteristics of the synthesized nanoformulae.

Trial Number	Sericin Percentage (%)	Stirring Time (min)	Physical Characteristics		
			Zeta Potential (mV)	Size (nm)	PDI
1	30.0	15.0	32.7	268.0	0.30
2	30.0	30.0	23.7	234.0	0.36
3	30.0	45.0	39.3	116.0	0.24
4	30.0	60.0	24.4	222.6	0.32
5	40.0	15.0	20.5	762.8	0.76
6	40.0	30.0	13.4	1438.0	0.97
7	40.0	45.0	33.3	143.2	0.27
8	40.0	60.0	20.4	642.3	0.75
9	50.0	15.0	35.5	629.6	0.65
10	50.0	30.0	27.5	166.1	0.28
11	50.0	45.0	35.6	169.3	0.27
12	50.0	60.0	28.2	7540.0	1.00
13	60.0	15.0	28.6	389.6	0.45
14	60.0	30.0	23.0	362.9	0.39
15	60.0	45.0	23.6	617.3	0.71
16	60.0	60.0	24.8	375.0	0.36
17	70.0	15.0	25.7	319.1	0.39
18	70.0	30.0	23.0	270.3	0.21
19	70.0	45.0	22.7	269.9	0.37
20	70.0	60.0	27.2	394.4	0.46

**Table 4:** Optimization of the antibacterial activity of the synthesized nanoformulae against several pathogens.

Trial Number	Sericin Percentage (%)	Stirring Time (min)	Inhibition Zone Diameter (mm)									
			*S. aureus* 1	*S. aureus* 2	*K. pneumoniae* 1	*K. pneumoniae* 2	*E. coli* 1	*E. coli* 2	*P. aeruginosa* 1	*P. aeruginosa* 2	*A. baumannii* 1	*A. baumannii* 2
1	30.0	15.0	18.0	11.0	8.0	6.0	6.0	13.0	6.0	6.0	15.0	11.0
2	30.0	30.0	24.0	12.0	8.0	7.0	6.0	8.0	6.0	6.0	15.0	12.0
3	30.0	45.0	32.0	22.0	12.0	9.0	9.0	14.0	8.0	12.0	15.0	12.0
4	30.0	60.0	26.0	21.0	12.0	6.0	6.0	6.0	6.0	6.0	14.0	12.0
5	40.0	15.0	12.0	6.0	6.0	6.0	7.0	8.0	6.0	6.0	10.0	6.0
6	40.0	30.0	8.0	6.0	8.0	6.0	9.0	10.0	6.0	6.0	8.0	6.0
7	40.0	45.0	12.0	6.0	8.0	6.0	6.0	11.0	6.0	6.0	6.0	6.0
8	40.0	60.0	18.0	10.0	6.0	6.0	6.0	6.0	6.0	6.0	13.0	11.0
9	50.0	15.0	14.0	14.0	6.0	6.0	6.0	6.0	6.0	6.0	11.0	11.0
10	50.0	30.0	14.0	12.0	6.0	6.0	6.0	6.0	6.0	6.0	9.0	6.0
11	50.0	45.0	18.0	17.0	8.0	6.0	6.0	6.0	6.0	6.0	12.0	6.0
12	50.0	60.0	12.0	10.0	8.0	6.0	6.0	6.0	6.0	6.0	8.0	8.0
13	60.0	15.0	6.0	6.0	6.0	6.0	6.0	6.0	6.0	6.0	6.0	6.0
14	60.0	30.0	10.0	6.0	8.0	8.0	6.0	6.0	6.0	6.0	6.0	6.0
15	60.0	45.0	10.0	6.0	10.0	6.0	6.0	6.0	8.0	8.0	7.0	7.0
16	60.0	60.0	6.0	6.0	8.0	6.0	6.0	6.0	6.0	7.0	6.0	6.0
17	70.0	15.0	10.0	6.0	7.0	6.0	6.0	6.0	8.0	6.0	6.0	6.0
18	70.0	30.0	12.0	10.0	6.0	6.0	6.0	6.0	6.0	11.0	6.0	6.0
19	70.0	45.0	10.0	6.0	6.0	6.0	6.0	8.0	6.0	8.0	6.0	6.0
20	70.0	60.0	6.0	6.0	6.0	6.0	7.0	7.0	6.0	6.0	6.0	6.0

*S. aureus*: *Staphylococcus aureus*, *K. pneumoniae*: *Klebsiella pneumoniae*, *E. coli*: *Escherichia coli*, *P. aeruginosa*: *Pseudomonas aeruginosa* and *A. baumannii*: *Acinetobacter baumannii*.

**Table 5:** The combined action between the tested antibiotics and the prepared nanoformulae.

Treatment/M.O_S_	Inhibition Zone Diameter (mm)				
	*Staphylococcus aureus*	*Klebsiella pneumoniae*	*Escherichia coli*	*Pseudomonas aeruginosa*	*Acinetobacter baumannii*
Sericin	8.0	8.0	7.5	7.0	7.5
Propolis	7.5	8.0	7.0	7.0	7.0
nSE/P	22.0	9.0	9.0	8.0	12.0
Cephalexin	11.7	12.5	10.5	9.5	10.0
nSE/P/Cephalexin	45.0	30.0	25.5	24.5	25.3
Colistin	10.0	11.0	9.0	8.6	9.0
nSE/P/Colistin	22.0	27.5	19.0	18.0	19.0
Amoxicillin	11.0	12.0	9.7	9.0	9.5
nSE/P/Amoxicillin	34.0	26.4	22.0	20.0	21.0
Tetracycline	6.0	6.0	6.0	6.0	6.0
nSE/P/Tetracycline	15.0	8.0	6.0.0	6.0	6.0
Tegycicline	22.0	17.0	25.0	17.0	14.0
nSE/P/Tegycicline	15.0	6.0	6.0	6.0	12.0
Chloramphenicol	25.0	20.0	6.0	6.0	7.0
nSE/P/Chloramphenicol	25.0	20.0	6.0	6.0	7.0
Ampicillin-sulbactam	16.0	6.0	13.0	6.0	10.0
nSE/P/Ampicillin-sulbactam	19.0	6.0	13.0	6.0	12.0
Cefotaxime	12.0	13.0	15.0	15.0	14.0
nSE/P/Cefotaxime	14.0	11.0	14.0	10.0	12.0
Cefuroxime	6.0	6.0	6.0	6.0	6.0
nSE/P/Cefuroxime	17.0	6.0	6.0	6.0	6.0
Gentamicin	23.0	22.0	10.0	10.0	8.0
nSE/P/Gentamicin	23.0	24.0	10.0	12.0	8.0
Cefoperazone	22.0	25.0	20.0	19.0	14.0
nSE/P/Cefoperazone	25.0	25.0	23.0	22.0	16.0
Azithromycin	6.0	15.0	13.0	6.0	12.0
nSE/P/Azithromycin	15.0	14.0	10.0	6.0	10.0
Ceftazidim	6.0	13.0	13.0	6.0	10.0
nSE/P/Ceftazidim	15.0	6.0	6.0	6.0	10.0
Amikacin	25.0	23.0	24.0	25.0	7.0
nSE/P/Amikacin	30.0	24.0	24.0	28.0	9.0

**Table 6:** MIC, MBC and MIC index evaluation of the most promising nanoformula against *P. aeruginosa*.

M.O_S_	*Pseudomonas aeruginosa*		
	MIC (µg/mL)	MBC (µg/mL)	MIC Index
Sericin	750.0	3000.0	4.0
Propolis	1250.0	5000.0	4.0
nSE/P	250.0	1000.0	4.0
nSE/P/Amoxicillin	1.0	4.0	4.0

**Table 7:** Independent variables and their level of variation.

Independent Variables	Levels of Variation				
	−2	−1	0	+1	+2
Sericin concentration (%)	30.0	40	50	60	70
Stirring time (min)	0.0	15	30	45	60

## Data Availability

Not applicable.

## Supplementary Materials

The following supporting information can be downloaded at: https://www.mdpi.com/1422-0067/23/19/11654/s1.
