# Correction: Diad et al. Novel Amoxicillin-Loaded Sericin Biopolymeric Nanoparticles: Synthesis, Optimization, Antibacterial and Wound Healing Activities. *Int. J. Mol. Sci.* 2022, *23*, 11654

**DOI:** 10.3390/ijms25136923

**Published:** 2024-06-25

**Authors:** Shaimaa E. Diab, Nourhan A. Tayea, Bassma H. Elwakil, Abir Abd El Mageid Gad, Doaa A. Ghareeb, Zakia A. Olama

**Affiliations:** 1 Botany and Microbiology Department, Faculty of Science, Alexandria University, Alexandria 21568, Egypt; 2 Medical Laboratory Technology Department, Faculty of Applied Health Sciences Technology, Pharos University in Alexandria, Alexandria 21500, Egypt; 3 Applied Entomology Department, Faculty of Agriculture, Alexandria University, Alexandria 21545, Egypt; 4 Biological Screening and Preclinical Trial Lab, Biochemistry Department, Faculty of Science, Alexandria University, Alexandria 21568, Egypt

In the original publication [1], there was a mistake in Figure 7 as published. There was disorganization in the pictures. The corrected Figure 7 appears below. The authors state that the scientific conclusions are unaffected. This correction was approved by the Academic Editor. The original publication has also been updated.

## Figures and Tables

**Figure 7:**
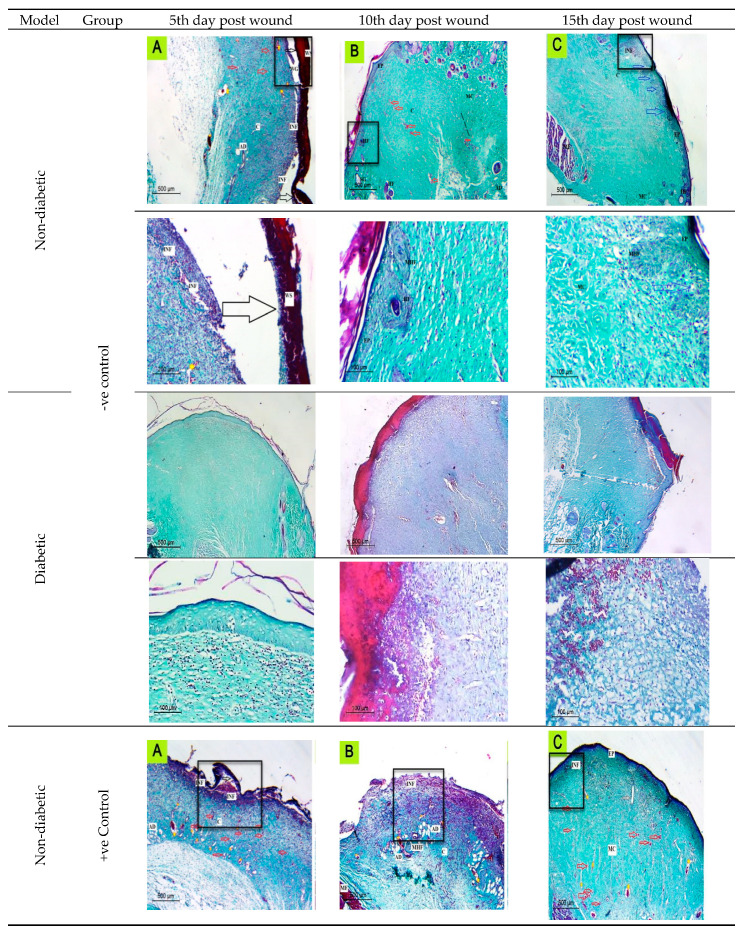

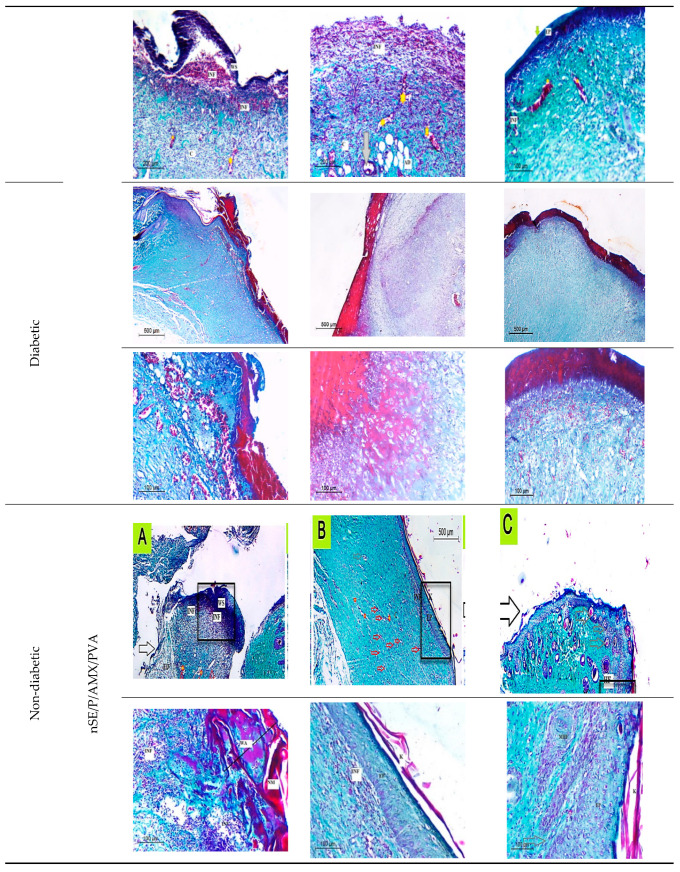

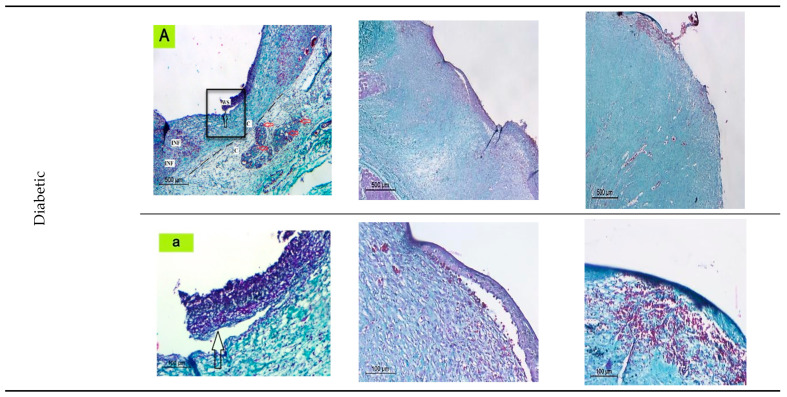
The histological evaluation of skin wounds at different time intervals (5th, 10th, and 15th) indicated regression of the lesions with better epithelialization (blue arrows) and more effective re-organization of the dermis by collagen fiber maturation. Inflammatory cells (INF); Adipose connective tissue; blood vessels (red arrows); Immature collagen (IMC); mature collagen (MC); epidermis (EP); maturating hair follicle (MHF); Wound Gap (WG); wound area (WA); wound scab (WS) dilated blood vessels (Yellow Strikes); Muscle Fibers (MF); detached scabs (black arrows).
