# Supplementary material for: Novel Amoxicillin-Loaded Sericin Biopolymeric Nanoparticles: Synthesis, Optimization, Antibacterial and Wound Healing Activities
Source: Int J Mol Sci. 2022 Oct 1;23(19):11654. doi: 10.3390/ijms231911654 (PMC9570309; doi:10.3390/ijms231911654)
Supplement: Supplementary file 1 [file ijms-23-11654-s001.zip › ijms-1875170-supplementary.pdf]

Novel amoxycillin loaded sericin biopolymeric nanoparticles: Synthesis, Optimization and antibacterial and wound healing activities

Shaimaa E. Diab[1], Nourhan A. Tayea[1], Bassma H. Elwakil[2*], Abir Abd El Mageid Gad[3], Doaa A. Ghareeb[4] and Zakia A. Olama[1]

[1] Botany and Microbiology Department, Faculty of Science, Alexandria University, Alexandria, Egypt
[2] Medical Laboratory Technology Department, Faculty of Applied Health Sciences Technology, Pharos University in Alexandria, Alexandria, Egypt
[3] Applied Entomology Department, Faculty of Agriculture, Alexandria University, Alexandria, Egypt
[4] Biological Screening and Preclinical Trial Lab, Biochemistry Department, Faculty of Science, Alexandria University, Alexandria, Egypt

Table S1. ANOVA study of the experimental responses.

Dependent variable	Zeta size (nm)				
	DF	Adj. SS	Adj. MS	F-value	p-value
**Corrected Model**	19	150097773.36	7899882.81	219644.564	<0.001*
**Ser. %**	4	29074812.14	7268703.04	202095.543	<0.001*
**St-Time (min)**	3	23248530.87	7749510.29	215463.678	<0.001*
**Ser. % * St-Time (min)**	12	97774430.35	8147869.20	226539.459	<0.001*
**Lack-of-fit**	0	0.000	–	–	–

	Zeta pot. (mV)				
	DF	Adj. SS	Adj. MS	F-value	p-value
**Corrected Model**	19	2185.768	115.040	3.381	0.001*
**Ser. %**	4	1090.506	272.627	8.012	<0.001*
**St-Time (min)**	3	431.537	143.846	4.227	0.011*
**Ser. % * St-Time (min)**	12	663.726	55.310	1.625	0.123
**Lack-of-fit**	0	0.000	–	–	–

	PDI				
	DF	Adj. SS	Adj. MS	F-value	p-value
**Corrected Model**	19	3.400	0.179	26.309	<0.001*
**Ser. %**	4	1.126	0.282	41.382	<0.001*
**St-Time (min)**	3	0.333	0.111	16.298	<0.001*
**Ser. % * St-Time (min)**	12	1.942	0.162	23.788	<0.001*
**Lack-of-fit**	0	0.000	–	–	–

	Antibacterial activity (mm)				
	DF	Adj. SS	Adj. MS	F-value	p-value
**Corrected Model**	19	375.495	19.763	22.988	<0.001*
**Ser. %**	4	332.477	83.119	96.685	<0.001*
**St-Time (min)**	3	11.439	3.813	4.435	0.009*
**Ser. % * St-Time (min)**	12	31.578	2.632	3.061	0.004*
**Lack-of-fit**	0	0.000	–	–	–

: Not applicable

Table S2. Regression model for independent variables.

Dependent variables	R^2 (%)	Adjusted R^2 (%)
**Zeta size (nm)**	7.5	4.3
**Zeta-pot. (mV)**	0.3	-3.2
**PDI**	1.0	-2.5
**Antibacterial (mm)**	62.8	61.5

Table S3. One-way ANOVA of the entrapment efficiency versus Amoxycillin conc (mg/mL).

Source	DF	Adj SS	Adj MS	F-Value	P-Value
Amoxycillin conc (mg/mL)	2	2972.41	1486.21	8917.24	0.000
Error	3	0.50	0.17	-	-
Total	5	2972.91	-	-	-

: Not applicable
